# The IL-4/-13 Axis and Its Blocking in the Treatment of Atopic Dermatitis

**DOI:** 10.3390/jcm11195633

**Published:** 2022-09-24

**Authors:** Georgia Pappa, Dimitrios Sgouros, Konstantinos Theodoropoulos, Antonios Kanelleas, Evangelia Bozi, Stamatios Gregoriou, Konstantinos Krasagakis, Alexander C. Katoulis

**Affiliations:** 1 2nd Department of Dermatology and Venereology, “Attikon” General University Hospital, Medical School, National and Kapodistrian University of Athens, 12462 Athens, Greece; 2 1st Department of Dermatology and Venereology, “Andreas Suggros” Hospital, Medical School, National and Kapodistrian University of Athens, 16121 Athens, Greece; 3 Department of Dermatology, University General Hospital of Heraklion, 71500 Heraklion, Greece

**Keywords:** atopic dermatitis, interleukin 4/13, Th2 response, treatment, biologics

## Abstract

Atopic dermatitis (AD) is a common inflammatory skin disease with a complex pathophysiology, intertwining immune dysregulation, epidermal barrier dysfunction, IgE sensitization, environmental factors and genetic predisposition. It has been recently identified that interleukins -4 and -13 play crucial roles in the type-2-driven inflammation that characterizes AD, contributing to its symptomatology. Novel therapeutic approaches that target Th2 cytokines and their respective pathways have been developed, aiming to optimize the treatment of AD.

## 1. Introduction

Atopic dermatitis (AD) is a chronic, pruritic inflammatory skin disease, affecting up to 25% of children and approximately 10% of adults, including 1–3% of the elderly [1,2]. It presents with variable clinical phenotypes, and it is primarily characterized by eczematous lesions, intense pruritus and xerosis [3].

The pathophysiology involves a complex interaction of immune dysregulation, epidermal barrier dysfunction and immunoglobulin (Ig)E sensitization on a genetic background, triggered by environmental factors [4,5]. The immune activation of the AD is characterized by robust type-2-driven inflammation, orchestrated by multiple cytokines, including, interleukins (IL)-4 and -13, which are regarded as pivotal to AD pathogenesis [5,6]. 

AD with its chronic relapsing course; its debilitating symptoms, including sleep disturbances; its comorbidities; and its psychosocial impact significantly impairs patients’ quality of life [7,8]. Despite its high prevalence and impact, there are unmet needs in the treatment of AD, especially for patients with moderate-to-severe AD [9].

In the present review, we discuss the role of the IL-4 and IL-13 in the pathogenetic cascade of AD. We also focus on emerging targeted therapies for the treatment of AD by blocking these pathways.

## 2. Interleukin-4 and Intereleukin-13

Current research depicts the pivotal role of both these Th2 cytokines in the pathogenesis of AD, as well as their key roles in the atopic march [10]. The genes encoding IL-4, IL-5, IL-13, and granulocyte-macrophage colony-stimulating factor (GM-CSF) are located in a cluster on chromosome 5 [11]. A variety of gene polymorphisms of both IL-4 and IL-13 and their receptors have been linked to a genetic predisposition for AD onset and development in both children and adults [12,13,14]. 

Both cytokines participate in multiple steps of the pathophysiologic cascade of AD. It is well known that they both use Janus kinases (JAKs) to initiate signaling and to activate signal transducer and activator of transcription-6 (STAT6), the main transcription factor for many of their biologic functions [15]. Previous reports have described IL-4 and IL-13 overexpression in acute and chronic skin lesions of patients with AD [16]. IL-4 promotes the differentiation of naïve CD4+ T cells into Th2 cells. The Th2 cells, in turn, stimulate the production of several inflammatory cytokines (IL-4, IL-13, IL-5 and IL-19), which are involved in the recruitment of eosinophils, basophils and mast cells and in the release of allergic mediators [17,18,19]. In addition, they induce the synthesis of IgE by B cells [20]. Moreover, IL-4, similarly induces the development of T cytotoxic (Tc)-2 cells and innate lymphoid cells (ILC)-2 cells, modulates dendritic cell activity and stimulates the release of IL-10 [19]. Thus, the differentiation of naïve CD4+ and CD8+ cells towards type 2 cells is further favored, potentiating type 2 inflammation [19]. This is in line with the finding of significant expansion of Th2 cell frequencies in cutaneous lymphocyte-associated antigen-positive (CLA+) T cells of adult patients with moderate-to-severe AD, compared with healthy controls [21]. IL-4 and IL-13 are also responsible for maintenance and amplification of Th2 cell recruitment by continuous production of Th2 cytokines and chemokines, such as eotaxins 1, 2 and 3 [22]. Recently, it was suggested that IL-4 and IL-13 promote the neurogenic itch, which is the hallmark of AD, via two non-histaminergic pathways: directly, by stimulating itch sensory neurons, and indirectly, via interaction with the IL-31 pathway, a well-known pruritogenic cytokine, through up-regulation of the IL-31 receptor in keratinocytes and dorsal root ganglia [23,24]. 

It is also well-documented that IL-4 and IL-13 contribute significantly in the skin barrier function impairment of AD [25]. Both these interleukins not only stimulate epidermal keratinocyte differentiation, but also significantly down-regulate the expression of important structural epidermal proteins (such as filaggrin, loricrin and involucrin) and alter the extracellular lipid composition, crucial for the normal epidermal barrier function [25,26]. Consequently, IL-4 and IL-13 contribute to the “leaky” skin and the increased transepidermal water loss that characterizes AD. Additionally, they attenuate the production of antimicrobial peptide (AMP) and suppress the up-regulation of innate immune response genes, such as β-defensins 2 and 3, LCN2 and cathelicidin LL-37, rendering the skin susceptible to infectious pathogens and leading to skin dysbiosis [27,28,29]. Interestingly, both IL-4 and IL-13 induce the synthesis of collagen and fibrinogen, which facilitate the adhesion of *Staphylococcus aureus* that commonly colonizes AD skin [29].

IL-13 holds a distinctive role that differentiates it from IL-4, inducing excessive production of collagen by fibroblasts, leading to increased collagen deposition and tissue remodeling [30]. Tissue remodeling is further promoted by IL-13-induced production of metalloproteinase (MMP)-9 in human keratinocytes, which facilitates the migration of inflammatory cells into the epidermis through the basement membrane [31]. In addition, IL-13 down-regulates MMP-13 in human dermal fibroblasts, resulting in decreased collagen degradation and fibrosis [31,32]. This process is clinically evident in the thickened, lichenified skin lesions of chronic AD patients [32]. 

## 3. Emerging Treatments for AD

Recent understanding of the crucial roles of IL-4 and IL-13 in multiple steps of the pathogenetic cascade of AD has led to the development of new targeted therapies. The distinct Th2-related immunologic hallmarks associated with IL-4 and IL-13 depend on the varying expression of the type-I and type-II IL-4 receptors on cells. The common gamma chain when combined with IL-4Rα forms the type-I receptor, while when combined with IL-13Rα, forms the type-II receptor. IL-4, by binding to IL-4Rα, can bind to both receptor types. In contrast, IL-13, by binding to IL-13RαI, can only bind to the type-II receptor [33]. It seems that IL-4 functions centrally, via type I receptors expressed in the lymph nodes, orchestrating the type 2 response by promoting T cell and ILC differentiation [33]. On the other hand, IL-13 acts more peripherally, at the tissue level, via the type II receptor and YKL-40 (chitinase-3-like protein 1) [33]. Hence, dual blockade of both IL-4 and IL-13 leads to a quick but also maintained treatment result [33]. On the contrary, a blockade of IL-4 signaling solely, may cause a delayed therapeutic response [34].

## 4. IL-4 Inhibitors

### 4.1. Dupilumab

Dupilumab is a fully human monoclonal antibody that acts by blocking the IL-4Rα, thereby inhibiting the activity of both IL-13 and IL-4 downstream signaling, through a blockade of their common IL-4α subunit [35]. It is the first approved monoclonal antibody for the treatment of adults with moderate-to-severe AD [35,36]. The approved treatment regimen for adults is 600 mg subcutaneously once and, then, 300 mg every other week [35]. More recently, it has also been approved for children ≥6 months old, with the pediatric dosage being body weight-dependent [37]. Its approval was based on the efficacy results demonstrated across multiple trials by significantly improving moderate-to-severe AD, as measured by SCORing Atopic Dermatitis (SCORAD), Eczema Area and Severity Index (EASI), Investigator Global Assessment (IGA) severity scores, and pruritus Visual Analogue Scale (VAS) [38]. Data from a recent real-world study in adults with moderate-to-severe AD treated with dupilumab showed statistically significant improvements in IGA score [39]. Seventy percent achieved IGA score ≤ 2 and 42.8% IGA 0/1, from baseline IGA ≥ 3, at 4 months [39]. Statistically significant reductions in the mean values of patient-reported pruritus severity were observed, as well as in the mean values of body surface area from baseline to 4 months (7.0 vs. 2.8 and 39.3% vs. 16.3%, respectively) [39].

A combination of dupilumab with topical therapy has been proven to be even more efficacious. A one-year phase III study (LIBERTY AD CHRONOS) showed superior results in EASI-75 and peak pruritus numerical rating scale (P-NRS) scores in patients treated with dupilumab and topical corticosteroids (TCs) or calcineurin inhibitors, compared to the results of dupilumab monotherapy clinical trials (SOLO1 and SOLO2) [40]. 

Interestingly, data from four phase 3 trials pointed out that patients with coexisting AD, asthma and sino-nasal conditions, treated with dupilumab, showed important clinical improvement in all three allergic conditions [41]. Therefore, dupilumab may be the treatment of choice in patients suffering from multiple type 2 comorbid conditions of the atopic march [41].

Adverse events (AEs) related to dupilumab have been reported to be mild-to-moderate in severity and transient. The most commonly reported AEs include injection site reactions, conjunctivitis, and eosinophilia [42]. The principal pathogenetic mechanism of the dupilumab-induced eosinophilia is the inhibition of IL-4/IL-13-driven eosinophil migration from the circulation into the periphery [43]. Physiologically, these cytokines mediate the expression of factors that participate in the chemotaxis of eosinophils [43]. This paradoxical eosinophilia is also thought to be responsible for the allergic conjunctivitis associated frequently with dupilumab [44]. Real life data from the use of dupilumab in various clinical settings have described AEs not previously mentioned, such as paradoxical head and neck erythema, psoriasiform dermatitis, new-onset seronegative inflammatory arthritis and enthesitis, and new onset or worsening of alopecia areata [45].

### 4.2. Pitrakinra

Pitrakinra is an inactive recombinant human IL-4 mutein that specifically binds to IL-4Rα, preventing the downstream signaling of both IL-4 and IL-13 [46]. Although dupilumab also averts IL-4 and IL-13 signaling, the two agents differ significantly in their mechanisms of action. Pitrakinra antagonizes the activation of IL4-Rα, while dupilumab targets IL-4Rα, inhibiting its dimerization with IL-13Rα1 or the γ chain [47]. It was initially developed for the treatment of asthma, where it demonstrated good clinical results, encouraging its further use in the treatment of AD [48]. Pitrakinra was evaluated for the treatment of severe AD in a randomized, placebo-controlled phase II clinical trial [49]. Twenty-five patients received pitrankinra 30 mg subcutaneously, twice daily for 28 days. The results from this trial have not been published as yet [49].

### 4.3. Others

The clinical programs of two novel targeted anti-IL-4Rα monoclonal antibodies are currently under evaluation for the treatment of AD. CBP-201 is currently investigated in a phase IIb clinical trial, and AK120 is under a phase II placebo-controlled clinical trial [50,51].

## 5. IL-13 Inhibitors

### 5.1. Lebrikizumab

Lebrikizumab is a humanized IgG4 anti-IL-13 monoclonal antibody that acts by preventing the binding and heterodimerization of IL-13Rα1 and IL-4Rα, thus inhibiting downstream signaling transduction by IL-13 [52]. 

In a randomized, placebo-controlled phase II trial, for moderate-to-severe AD, lebrikizumab showed superior results, when compared to TCs (triamcinolone acetonide 0.1% and/or hydrocortisone 2.5%) applied twice daily [53]. At week 12, EASI-50 was achieved in 82% of patients treated with lebrikizumab vs. 62% in the placebo group, while IGA of 0/1 was reached in 33% of patients in the lebrikizumab treatment group compared with 19% in the placebo group [53]. In a phase IIb double-blind placebo-controlled trial, lebrikizumab administered over a period of 16 weeks, showed statistically significant dose-dependent improvement in EASI score in all three dose groups (125 mg every 4 weeks with a 250 mg loading dose, 250 mg every 4 weeks with a 500 mg loading dose and 250 mg every 2 weeks with a 500 mg loading dose at baseline and week 2) from baseline, compared to the placebo (62.3%, 69.2% and 72.1%, respectively vs. 41.1% in placebo) [54]. Furthermore, EASI-50, EASI-75, EASI-90, IGA of 0/1 scores and BSA were significantly improved in both lebrikizumab 250 mg treatment groups [54]. The therapeutic results are similar to those of dupilumab, with the additional advantage of less frequent dosing. Nevertheless, further research is required [54]. 

The safety profile of the drug is favorable, with only mild/moderate and transient AEs. Most commonly reported AEs were infections, including skin and upper respiratory tract infections, nasopharyngitis, headache, injection site pain and injection site reaction. All AEs were well tolerated by patients [53].

Currently, there is one ongoing phase II and eight phase III clinical trials of lebrikizumab in the treatment of moderate-to-severe AD [55,56]. Preliminary results suggest that combination therapy with steroids may significantly improve AD [55,56].

### 5.2. Tralokinumab

Tralokinumab is a fully human IgG4 monoclonal antibody that acts as an IL-13 inhibitor, by preventing IL-13 binding to IL-13Rα1 and IL-13Rα2 [57]. This differentiates it from lebrikizumab, which neutralizes IL-13 activity on IL-13Rα1 and IL-4Rα [57]. Tralokinumab was approved by the United States Food and Drug Administration (FDA) for the treatment of moderate-to-severe AD in 2021 [58]. The approved treatment regimen is a loading dose of 600 mg, administered subcutaneously, followed by 300 mg every other week [58]. The approval was based on data from three randomized double-blind, placebo-controlled phase III trials (ECZTRA 1–3) [59]. All aforementioned trials assessed tralokinumab 300 mg every other week (after a loading dose of 600 mg) either as monotherapy for 52 weeks (ECZTRA 1 and 2) or in combination with TCs as needed, for 32 weeks (ECZTRA 3) [59]. The results from all three trials met the co-primary endpoints of an IGA score of 0/1 and EASI-75 at week 16 [59]. More specifically, of the patients receiving tralokinumab, 15.8% achieved IGA of 0/1 and 25% achieved EASI-75 at week 16, compared to 7.1% and 12.7% in the placebo group, respectively (ECZTRA 1) [59]. In ECZTRA 2, 21% of patients that received tralokinumab improved to IGA of 0/1 and 33% achieved EASI-75 at week 16, compared to 9% and 10% in the placebo group, respectively [59]. In ECTZRA 3, the combination therapy with TCs proved to be even more efficacious, since 38% of patients treated with tralokinumab achieved IGA of 0/1 and 56% achieved EASI-75 at week 16, compared to 27% and 37% in the placebo group, respectively [60].

A meta-analysis that included five randomized, placebo-controlled trials of tralokinumab in adult moderate-to-severe AD reported that the most common AEs were upper respiratory tract infections, conjunctivitis, injection site reactions and eosinophilia [61].

### 5.3. Others

ASLAN004, a fully human monoclonal antibody targeting the IL-13Rα1 receptor, is currently under investigation for AD treatment [62]. Anrukinzumab (IMA-638) is an anti-IL-13 with a mechanism of action similar to that of lebrikizumab [63], while RPC4046 (ABT-308) and CNTO 5825 inhibit the binding of IL-13 to both IL-13Rα1 and IL-13Rα2 [64,65].

Table 1 summarizes all the emerging monoclonal antibodies for the treatment of AD, their mechanism of action and their most common AEs. 

## 6. Conclusions

AD is a chronic, multifaceted skin disease with a complex etiopathogenesis. It is well-established that the main pathophysiologic pathway of AD is the activation of the Th2 immune response, in which IL-4 and IL-13 play a central role, contributing significantly to AD signs and symptoms.

Advances in understanding IL-4/IL-13 pathway have opened the way for the development of novel therapeutic agents that target important steps of those signaling cascades. Most importantly, monoclonal antibodies that inhibit the IL-4/IL-13 pathway have revolutionized the treatment of moderate-to-severe AD. In this context, JAK inhibitors may constitute another successful addition, as JAK-STAT signaling interferes with the IL-4/IL-13 pathway. The ongoing scientific research raises hope for a more effective and safe, personalized management of AD.

## Figures and Tables

**Table 1:** Summary of IL-4 and IL-13 inhibitory monoclonal antibodies, their respective mechanism of action and their most common adverse events [42,50,53,61].

Monoclonal Antibody	Mechanism of Action	Common Adverse Events
Dupilumab	anti-IL-4Rα	Nasopharyngitis, upper respiratory tract infections, conjunctivitis, eosinophilia, injection-site reactions, exacerbation of AD
Pitrakinra	IL-13Rα1	Published data unavailable
CBP-201	anti-IL-4Rα	Headache, dizziness, upper respiratory tract infections
AK120	anti-IL-4Rα	Published data unavailable
Lebrikizumab	anti-IL-13	Injection site reactions, herpes virus infections, conjunctivitis
Tralokinumab	anti-IL-13	Upper respiratory tract infections, headaches conjunctivitis, injection site reactions, eosinophilia
ASLAN004	IL-13Rα1	Published data unavailable
Anrukinzumab (IMA-638)	anti-IL-13	Published data unavailable
RPC4046 (ABT-308)	IL-13Rα1 & IL-13Rα2	Published data unavailable
CNTO 5825	IL-13Rα1 & IL-13Rα2	Published data unavailable

AD, atopic dermatitis; IL, interleukin
